# High-fat diet-induced obesity impairs insulin signaling in lungs of allergen-challenged mice: Improvement by resveratrol

**DOI:** 10.1038/s41598-017-17558-w

**Published:** 2017-12-11

**Authors:** Diana M. André, Marina C. Calixto, Carolina Sollon, Eduardo C. Alexandre, Edith B. G. Tavares, Ana C. A. Naime, Gabriel F. Anhê, Edson Antunes

**Affiliations:** Department of Pharmacology, Faculty of Medical Sciences, University of Campinas (UNICAMP), Campinas, São Paulo Brazil

## Abstract

Insulin resistance plays an important role in obesity-associated asthma exacerbations. Using a murine model of allergic airway inflammation, we evaluated the insulin signaling transmission in lungs of obese compared with lean mice. We further evaluated the effects of the polyphenol resveratrol in the pulmonary insulin signaling. In lean mice, insulin stimulation significantly increased phosphorylations of AKT, insulin receptor substrate 1 (IRS-1) and insulin receptor β (IRβ) in lung tissue and isolated bronchi (p < 0.05), which were impaired in obese group. Instead, obese mice displayed increased tyrosine nitrations of AKT, IRβ and IRS-1 (p < 0.05). Two-week therapy of obese mice with resveratrol (100 mg/kg/day) restored insulin-stimulated AKT, IRS-1 and IRβ phosphorylations, and simultaneously blunted the tyrosine nitration of these proteins. Additionally, the c-Jun N-terminal kinase (JNK) and inhibitor of NF-κB Kinase (IκK) phosphorylations were significantly increased in obese group, an effect normalized by resveratrol. In separate experiments, the inducible nitric oxide synthase (iNOS) inhibitor aminoguanidine (20 mg/kg/day, three weeks) mimicked the protective effects exerted by resveratrol in lungs of obese mice. Lungs of obese mice display nitrosative-associated impairment of insulin signaling, which is reversed by resveratrol. Polyphenols may be putative drugs to attenuate asthma exacerbations in obese individuals.

## Introduction

Asthma is one of most frequent chronic respiratory disease characterized by variable symptoms of wheeze, shortness of breath, chest tightness and/or cough^1^. In spite of the activation of a myriad of cell types within the lungs of asthmatic individuals, the selective accumulation of eosinophils into the airways has assumed a central role of the asthma pathology^2^. Nitric oxide (NO) is a well-established proinflammatory mediator implicated in the eosinophilic airway inflammation, as evidenced in asthmatic patients^3^ and rodent models of asthma^4,5^. The inducible NO synthase (iNOS) enzyme is highly expressed in lung tissue of asthmatic patients and allergic animals, generating high levels of exhaled NO that positively correlates with the airway inflammation^3^.

Clinical and animal studies have documented a strong relationship between obesity and asthma aggravation^6^. Insulin-resistant obese mice fed a high-fat diet exhibited greater eosinophilic airway inflammation^7^ and bronchoconstriction^8^ upon allergen challenge in comparison with lean mice. Attenuation of the insulin resistance of obese mice by the anti-hyperglycemic drug metformin attenuates the eosinophilic airway inflammation and iNOS-derived NO concentrations to the levels of lean asthmatic group, suggesting that systemic insulin resistance play a role in asthma exacerbation in obesity conditions^9^. Additionally, prolonged administration of high-fat diet-fed mice with the iNOS inhibitor aminoguanidine fully restored the obesity-induced insulin resistance and significantly attenuated the eosinophil infiltration in the lung tissue^9^. Therefore, iNOS-derived NO may be a potential link between obesity-associated insulin resistance and asthma aggravation. Upregulation of iNOS has been clearly implicated in obesity and diabetes^10,11^, and NO overproduction induced by high-fat diet was reported to downregulate the insulin signaling in skeletal muscle, white adipose tissue and liver^12,13^. At the molecular level, NO-mediated nitrosative stress leads to covalent modifications thus inhibiting several key proteins that mediate insulin signaling^14,15^. Nevertheless, a greater understanding of the role of eosinophils in glucose homeostasis and in patients with asthma is warranted to provide more accurate phenotype-driven therapy^16^.

Resveratrol, a natural polyphenolic compound present in the skin of red grapes, improves glucose tolerance and insulin sensitivity^17–20^. Resveratrol also mimics the effect of calorie restriction on insulin-mediated glucose uptake in mouse skeletal muscles^21^ and stimulates the glucose uptake in diabetic rats^22^. Resveratrol also reduces adiposity by decreasing the accumulation of triglycerides in fat cells, which may in turns improve insulin resistance^23^. In high-fat diet-fed mice, two-week therapy with resveratrol reduced the fat mass and normalized the systemic insulin resistance, which was accompanied a large reduction of the eosinophilic airway inflammation in the antigen-challenged animals^24^. Lungs are highly vascularized, and insulin regulates migration, proliferation and inflammation in vascular smooth muscle cells^25^. We thought therefore that an impairment of insulin signaling in the lung tissues of obese mice would account for the exacerbation of allergic eosinophilic inflammation induced by high-fat diet, but no previous study has attempted to evaluate if insulin signaling transduction proteins exist in lung tissues. Therefore, in the present study, we first evaluated the phosphorylation of insulin signaling proteins in the lung tissues of high-fat diet fed obese compared with lean mice. We specifically evaluated the tyrosine phosphorylation of the β subunit of the insulin receptor (IRβ) and the insulin receptor substrate-1 (IRS-1), along with the serine phosphorylation of AKT enzyme, a serine/threonine kinase downstream IR crucial for the insulin signaling^26^. The phosphorylation of c-Jun N-terminal kinase (JNK) and Inhibitor of NF-κB Kinase (IκK), which are known to impair the insulin action^27^, was also evaluated. Second, we evaluated effects of resveratrol and aminoguanidine treatments in the insulin pathways (IR/AKT, JNK and IκK) in obese compared with lean mice.

## Results

### Resveratrol reduces the epididymal fat in obese mice

High-fat diet fed mice exhibited significant increases of body weight and epididymal fat mass compared with lean mice (p < 0.05). Table 1 shows that treatment with resveratrol in obese mice decreased by 12% and 21% body weight and epididymal fat, respectively (n = 5–6, p < 0.05). In lean group, resveratrol affected neither body weight nor epididymal fat mass (Table 1).

**Table 1:** Effects of treatment with resveratrol on body weight and epididymal fat. Mice fed a standard diet (lean group) or high-fat diet (obese group) were treated or not with resveratrol (Resv; 100 mg/kg/day, 2 weeks).

Groups	Body Weight (g)	Epididymal fat (g)
Lean	30.3 ± 0.008	0.25 ± 0.01
Obese	43.5 ± 0.008*	1.72 ± 0.12*
Lean + RESV	28.2 ± 0.008	0.27 ± 0.01
Obese + RESV	38.3 ± 0.008**	1.36 ± 0.08**

Data are expressed as mean ± SEM (n = 5–6). *p < 0.05 compared with lean group; **p < 0.05 compared with untreated obese group.

### Insulin-induced AKT phosphorylation is reduced in lungs and isolated bronchi of obese mice

**Figure 1:**
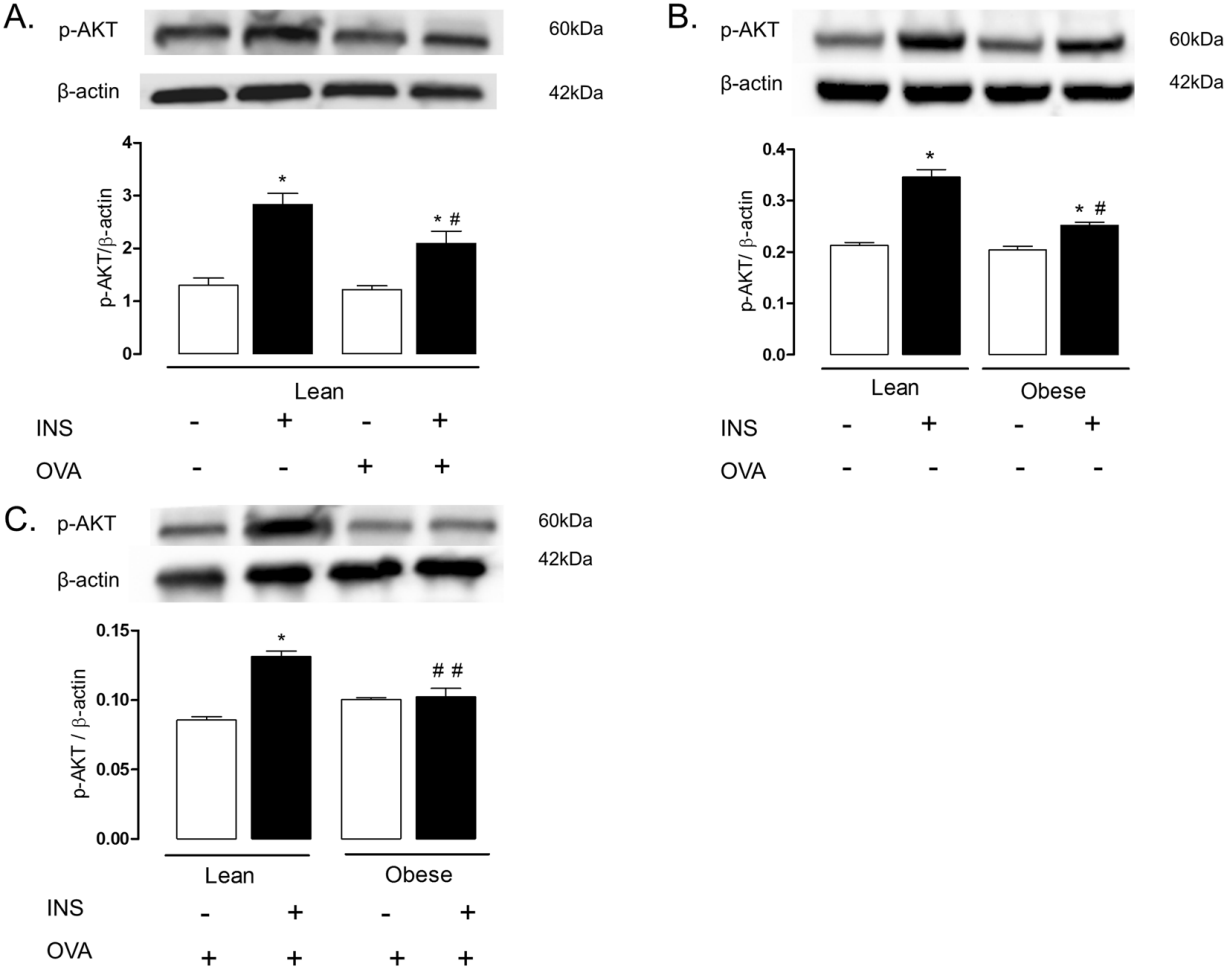
Expressions of phosphorylated AKT in the lungs of lean and/or high-fat diet-fed mice. Panel (A) indicates lungs of lean mice challenged or not with ovalbumin (OVA). Panel (B) indicates lungs of non-challenged obese compared with lean mice. Panel (C) indicates lungs of OVA-challenged obese compared with lean mice. Mice received an intravenous injection of insulin (INS; 1 U/animal), and within 5 min the lungs were processed. Phosphorylated AKT were normalized for β-actin. Data are expressed as mean ± SEM (*n* = 4–6). *p < 0.05 compared with respective basal (non-stimulated) group. ^#^p < 0.05 compared with insulin stimulation in lean group. ^##^p < 0.05 compared with insulin stimulation plus OVA challenge in lean group.

We initially evaluated the insulin-stimulated phosphorylation of AKT in lung tissue of non-challenged (instilled with saline) compared with OVA-challenged mice. In non-challenged mice, insulin stimulation significantly increased the AKT phosphorylation compared with basal levels (p < 0.05). In OVA-challenged mice, insulin stimulation also significantly elevated the AKT phosphorylation, but the phosphorylation level was lower than non-challenged group (p < 0.05; Fig. 1A).

Next, we evaluated the influence of obesity per se on insulin-stimulated phosphorylation of AKT in lung tissue in absence of allergic airway inflammation (saline-instilled mice). Our data showed that AKT phosphorylation by insulin was markedly reduced in lung tissue of obese compared with lean group, but still elevated compared with basal levels (p < 0.05; Fig. 1B). Finally, the insulin-induced AKT phosphorylation was investigated in lung tissues of OVA-challenged obese and lean mice. Data showed that AKT phosphorylation was nearly suppressed in OVA-challenged obese compared with lean mice ((p < 0.05; Fig. 1C).

For the further experiments addressing to characterize the insulin signaling proteins in lung tissues, independent groups of lean and obese mice were subjected to both OVA-challenge and stimulation with insulin. The phosphorylation of insulin signaling proteins in the lungs were also evaluated in lean and obese mice treated orally with resveratrol (100 mg/kg/day, two weeks).

### Resveratrol treatment restores the AKT phosphorylation in lung tissue and isolated bronchi of obese mice

**Figure 2:**
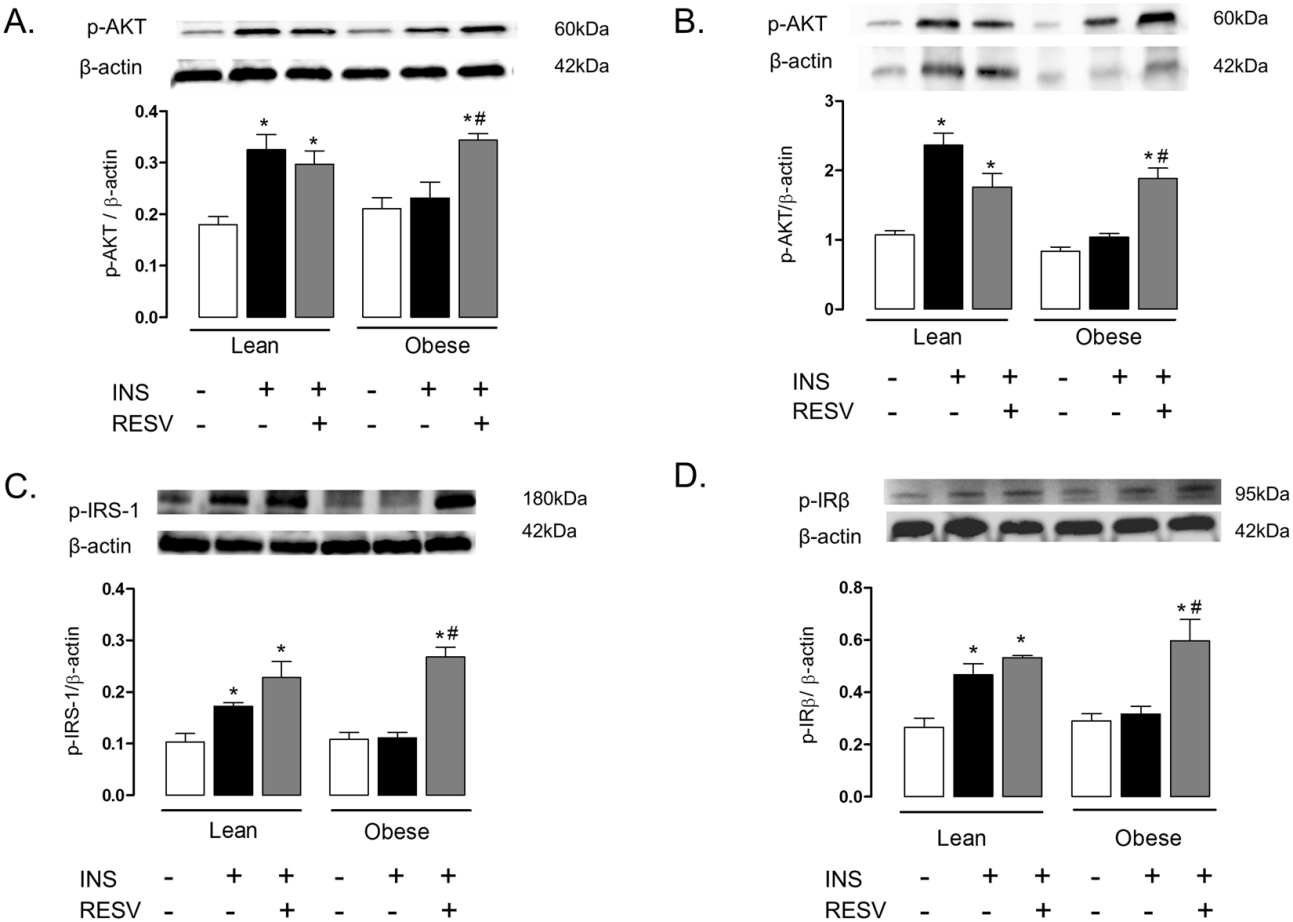
Expressions of phosphorylated AKT (panels A and B), IRS-1 (**C**) and IRβ (**D**) in lean and high-fat diet-fed mice, treated or not with resveratrol (Resv; 100 mg/kg/day, 2 weeks). Phosphorylated AKT is shown in lung tissue and isolated bronchi (panels A and B, respectively). Phosphorylated IRS-1 and IRβ in lung tissues are shown in panels (**C** and **D**), respectively. Mice received an intravenous injection of insulin (INS; 1 U/animal), and within 5 min the lungs were processed. Phosphorylated proteins were normalized for β-actin. Data are expressed as mean ± SEM (*n* = 6). *p < 0.05 compared with respective basal (non-stimulated) group. ^#^p < 0.05 compared with untreated obese group.

Figure 2A shows that insulin stimulation failed to increase AKT phosphorylation in lung tissue and isolated bronchi of obese mice as compared with lean group (p < 0.05; n = 6). Resveratrol treatment significantly enhanced AKT phosphorylation in both lung tissues (Fig. 2A) and isolated bronchi (Fig. 2B) of obese mice to similar levels of lean mice (n = 6). In lean group, resveratrol treatment did not affect significantly the AKT phosphorylation in lung tissue and isolated bronchi (Fig. 2A,B).

### Insulin-induced IRS-1 and IRβ phosphorylation are decreased in lung of obese mice

In lung tissues of lean mice, stimulation with insulin significantly increased IRS-1 (Fig. 2C) and IRβ (Fig. 2D) phosphorylation compared with non-stimulated lungs (p < 0.05, n = 6). However, in lung tissues of obese mice, insulin stimulation failed to increase the phosphorylation of IRS-1 and IRβ. Resveratrol treatment significantly elevated the phosphorylation of both of these proteins to the levels of the lean group (Fig. 2C,D). In lean mice, resveratrol treatment had no effect on insulin-induced phosphorylation of IRS-1 and IRβ.

### Nitration of proteins that mediate the insulin signaling is increased in lung tissue of obese mice

**Figure 3:**
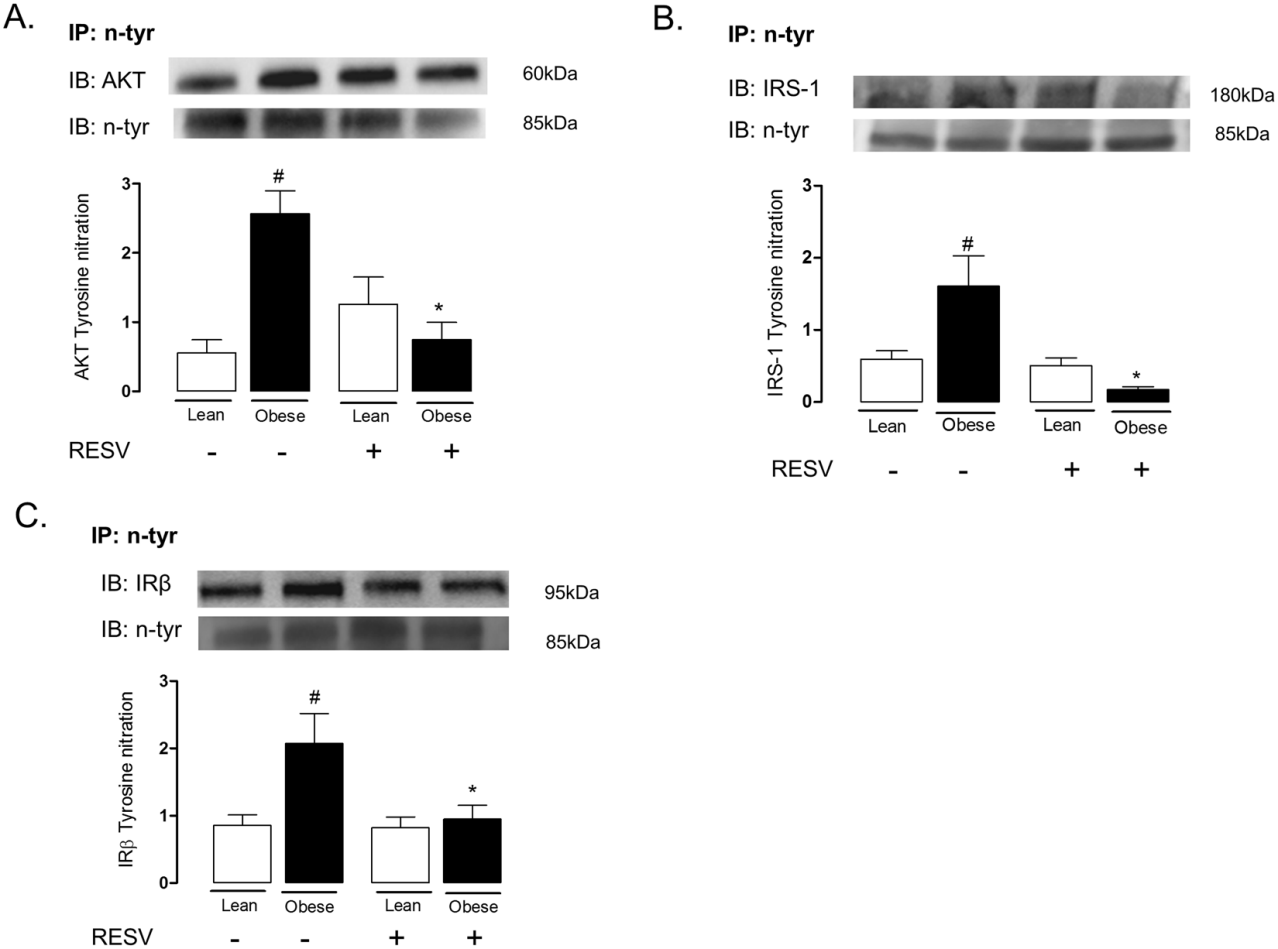
Immunoprecipitation of N-tyrosine and immunoblotting of AKT **(A)**, IRS-1 (**B**) and IRβ (**C**) in lung tissue of lean and high-fat diet-fed mice, treated or not with resveratrol (Resv; 100 mg/kg/day, 2 weeks). Immunoprecipitated AKT, IRβ and IRS-1 were normalized for immunoblotting of N-tyrosine. Data are expressed as mean ± SEM of 6 mice in each group. ^#^p < 0.05 compared with untreated lean mice. *p < 0.05 compared with untreated obese mice.

We next performed immunoprecipitation and immunoblotting assays to determine tyrosine nitration of AKT (Fig. 3A), IRS-1 (Fig. 3B) and IRβ (Fig. 3C) in lung tissue of obese and lean mice subjected to treatment with resveratrol. The tyrosine nitration of AKT, IRS-1 and IRβ were markedly increased in lung of obese mice as compared to those of the lean group (p < 0.05; n = 6). This modulation detected in the obese mice was fully normalized by resveratrol treatment. In the lean group, resveratrol treatment did not significantly affected AKT tyrosine nitration.

**Figure 4:**
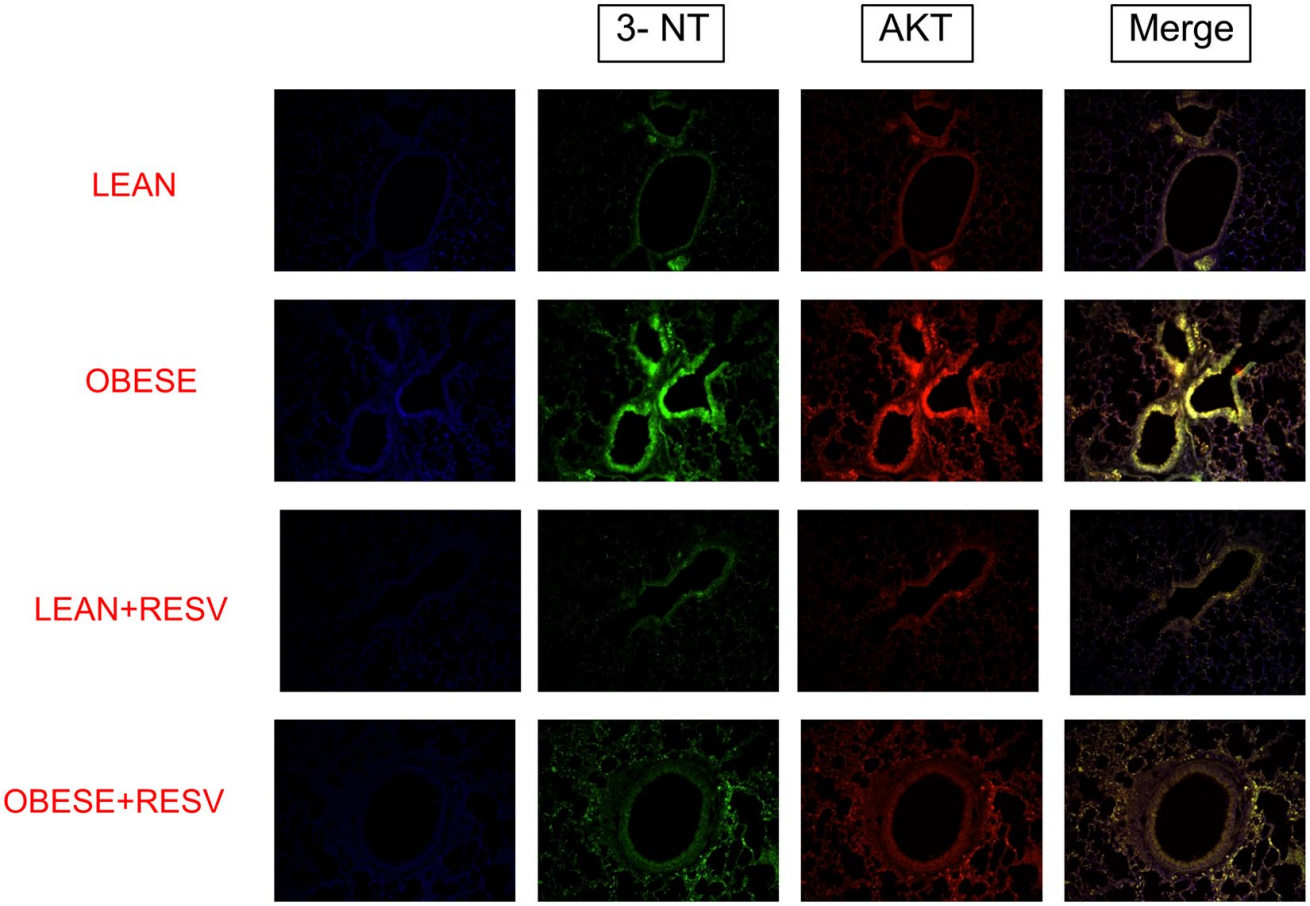
Immunofluorescence staining for tyrosine nitration of AKT in lung tissue of lean and high-fat diet-fed mice, treated or not with resveratrol (Resv; 100 mg/kg/day, 2 weeks). Representative immunostained DAPI (blue light), 3-NT (FITC green light) and AKT (rhodamine red)-conjugated secondary antibody with the two color images merged to demonstrate co-localization of tyrosine nitration of AKT (200× magnification).

**Figure 5:**
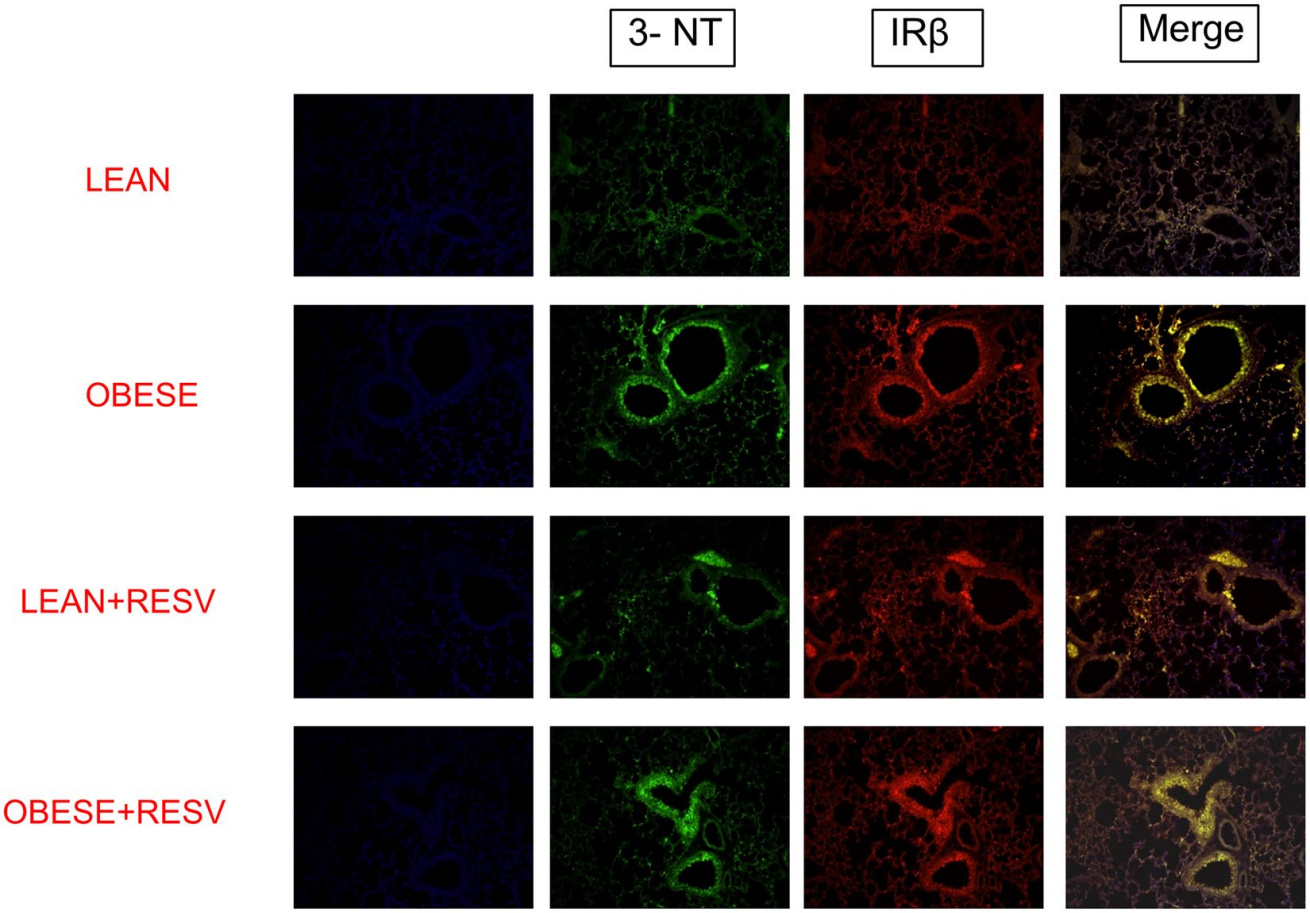
Immunofluorescence staining for tyrosine nitration of IRβ in lung tissue of lean and high-fat diet-fed mice, treated or not with resveratrol (Resv; 100 mg/kg/day, 2 weeks). Representative immunostained DAPI (blue light), 3-NT (FITC green light) and IRβ (rhodamine red)-conjugated secondary antibody with the two color images merged to demonstrate co-localization of tyrosine nitration of IRβ (200× magnification).

Immunofluorescence staining was performed to evaluate the nitration of the AKT and IRβ. The lung tissues of obese mice displayed increased tyrosine nitration of both AKT (Fig. 4) and IRβ (Fig. 5) as compared to those of the lean group (n = 6). These modulations were blunted by resveratrol treatment.

### JNK and IκK phosphorylation are elevated in lung tissue of obese mice

**Figure 6:**
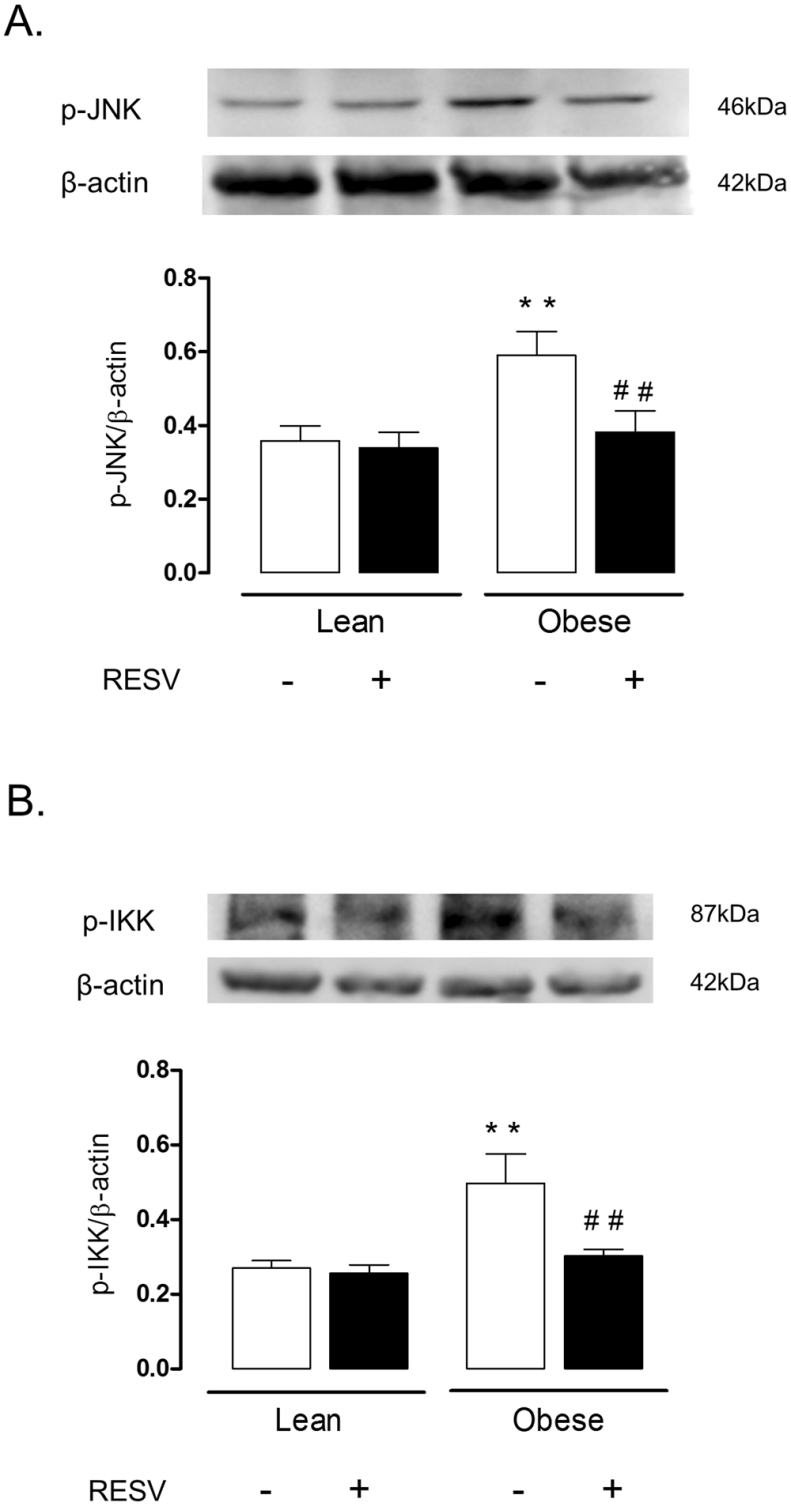
Expressions of p-JNK and p-IκK expressions in lung tissues of lean and high-fat diet-fed mice, treated or not with resveratrol (Resv; 100 mg/kg/day, 2 weeks). Phosphorylated JNK and IκK were normalized for β-actin. Data are expressed as mean ± SEM of 6 mice in each group. **p < 0.05 compared with untreated lean mice; ^##^p < 0.05 compared with untreated obese mice.

The phosphorylation of JNK and IκK was significantly greater in the lung tissue of obese as compared with those of lean mice (Fig. 6). Resveratrol treatment reduced the phosphorylation of these proteins in obese mice (n = 6).

### Aminoguanidine restores insulin signaling in lung tissue of obese mice

**Figure 7:**
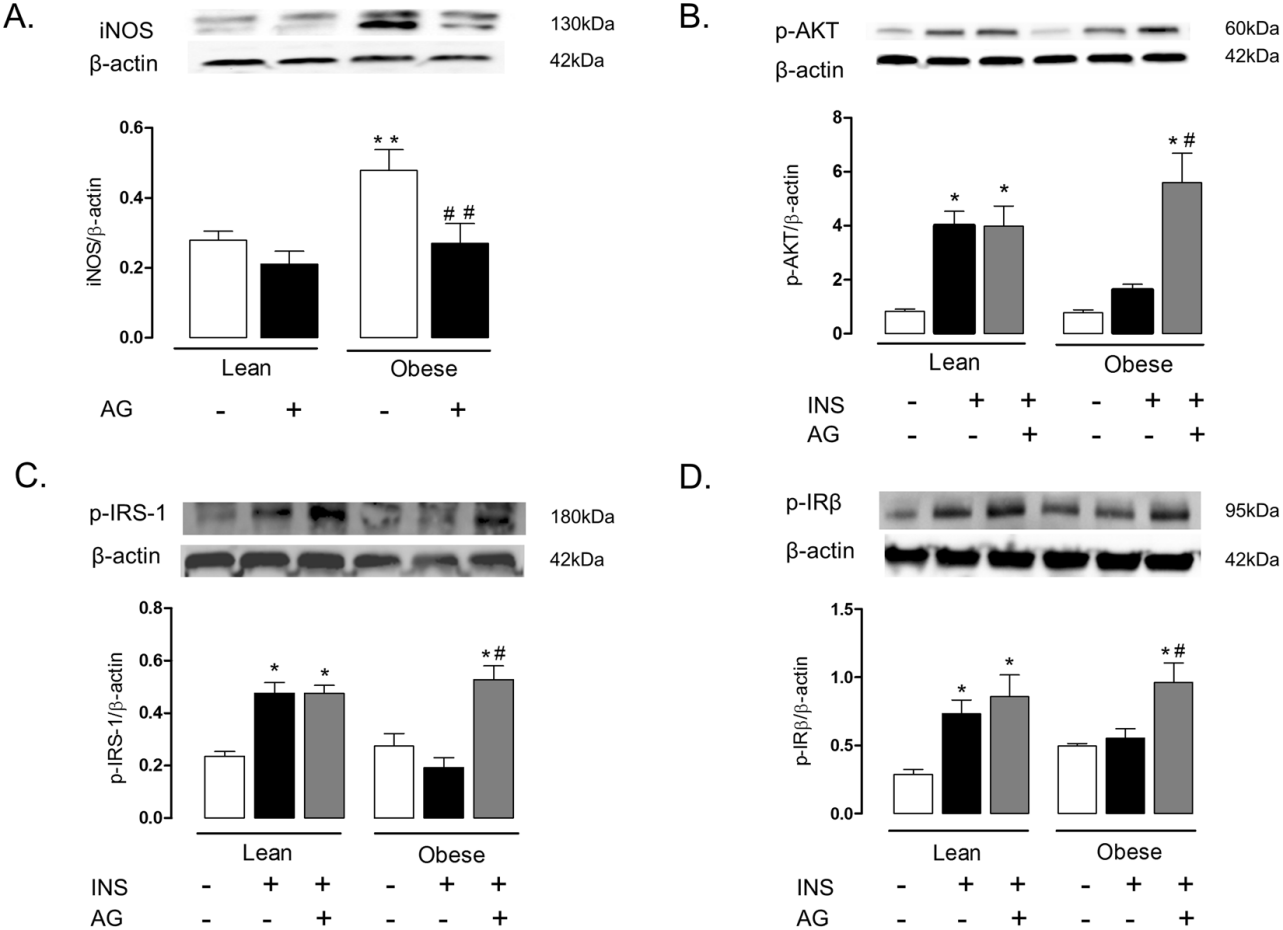
Expressions of inducible nitric oxide synthase (iNOS; **A**), phosphorylated AKT (**B**), IRS-1 (**C**) and IRβ (**D**) in lung tissues lean and high-fat diet-fed mice, treated or not with aminoguanidine (AG; 20 mg/kg/day, 3 weeks). Mice received an intravenous injection of insulin (INS; 1 U/animal), and within 5 min the lungs were processed. Phosphorylated AKT, IRS-1 AND IRβ and iNOS were normalized for β-actin. Data are expressed as mean ± SEM (*n* = 6). *p < 0.05 compared with respective basal (non-stimulated) group; ^#^p < 0.05 compared with untreated obese group; **p < 0.05 compared with lean untreated mice; ^##^p < 0.05 compared with obese untreated mice.

**Figure 8:**
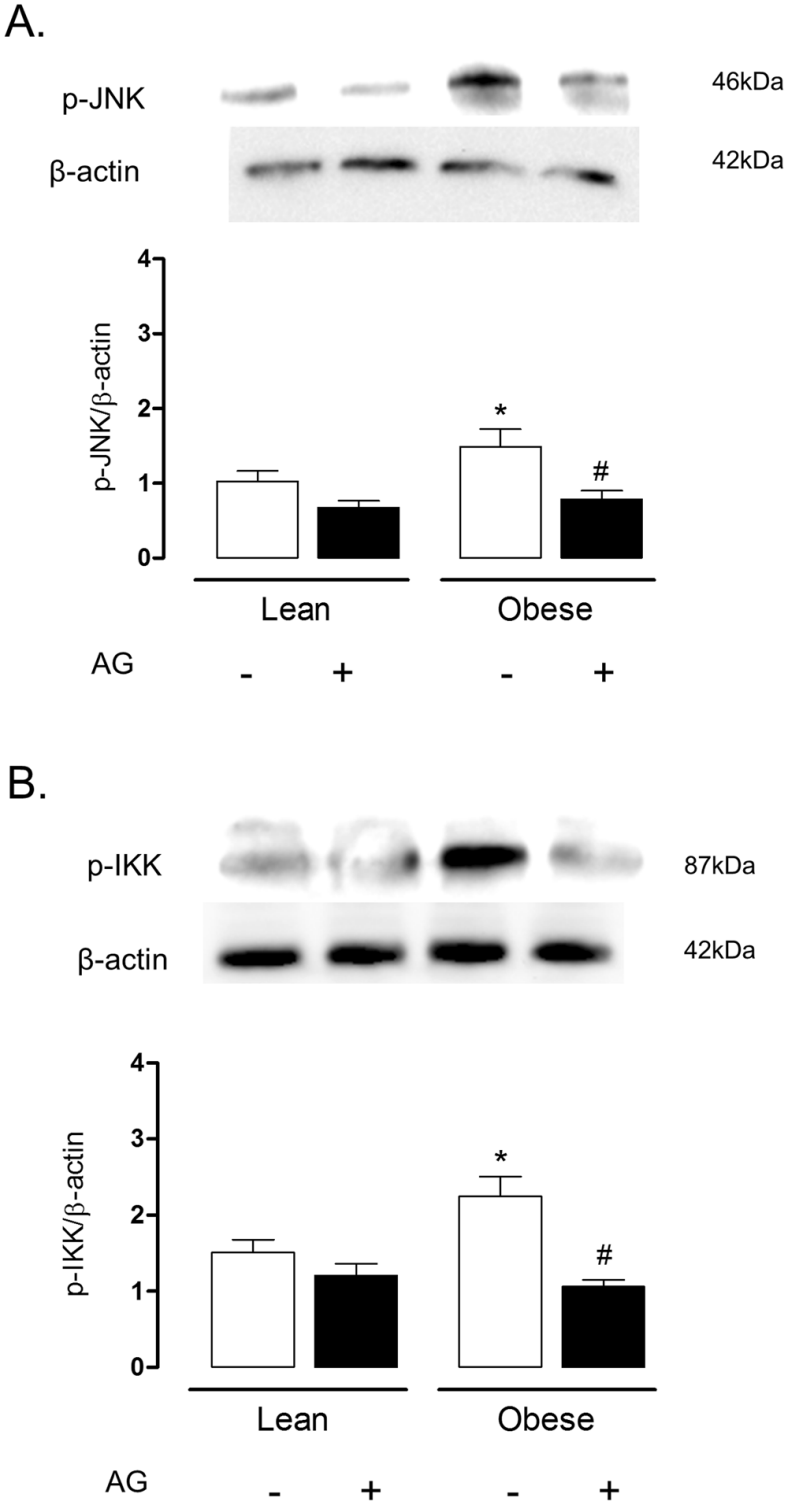
Expressions of p-JNK and p-IκK expressions in lung tissues of lean and high-fat diet-fed mice, treated or not with aminoguanidine (AG; 20 mg/kg/day, 3 weeks). Phosphorylated JNK and IκK were normalized for β-actin. Data are expressed as mean ± SEM of 6 mice in each group. *p < 0.05 compared with untreated lean mice; ^#^p < 0.05 compared with untreated obese mice.

In a separate set of animals, lean and obese mice were treated orally with the iNOS inhibitor aminoguanidine (20 mg/kg/day, three weeks), after which  the iNOS expression (Fig. 7A) and the phosphorylation of insulin-induced AKT, IRS-1 and IRβ were analyzed. Lung tissue of obese mice treated with aminoguanidine had significantly higher insulin-stimulated phosphorylation of AKT, IRS-1 and IRβ as compared with those of untreated mice (Fig. 7 B–D). In lung tissues of lean mice, aminoguanidine by itself had no effect on phosphorylation of AKT, IRS-1 and IRβ (Fig. 7B–D). In addition, aminoguanidine treatment blunted the increase in the phosphorylation of JNK and IκK in lung tissues induced by obesity (Fig. 8). With the dose used above, aminoguanidine restored iNOS expression in lung tissue of obese mice to those seen in the lean group (Fig. 7A).

## Discussion

Clinical studies have confirmed the association between asthma and obesity^28–32^. The higher prevalence of insulin resistance in obese and asthmatic patients compared with obese non-asthmatics has suggested that insulin resistance plays a role in obesity-related asthma exacerbation. However, no previous study has evaluated the insulin signal transduction in the lungs of obese asthmatic individuals. In the present study we used high-fat diet-fed mice to evaluate the insulin signaling in the lungs. Our present findings clearly indicate impaired insulin signaling in the lungs of obese mice, as evaluated by the reduced insulin-stimulated phosphorylation of AKT, IRS-1 and IR1β, along with increased phosphorylation of JNK and IκK.

In conditions of insulin resistance, insulin signaling is impaired at multiple levels, resulting in reduced glucose uptake by insulin-sensitive peripheral tissues^33^. AKT (also referred as PKB) is a serine/threonine kinase that plays a crucial role in insulin action by regulating insulin-stimulated translocation of GLUT4-enriched vesicles in muscle and adipose tissue^34^. Accordingly, AKT phosphorylation is reduced in type 2 diabetes patients^35^ and mice^36,37^. We describe here that insulin markedly increases AKT phosphorylation in lung tissue of lean but not in obese mice, which indicates that this tissue becomes unresponsive to insulin after 12 weeks of a high-fat diet. Similar data were collected from isolated bronchi, where insulin failed to increase the p-AKT phosphorylation in the obese group. Moreover, in lung tissue of lean mice, insulin stimulation significantly increased IRS-1 and IRβ phosphorylation, which was not observed in obese group. Altogether, these findings clearly show that lungs of high-fat diet-fed obese mice exhibit an impaired insulin action. A recent study has shown that human airway cells have functional insulin receptors, and that insulin dependent glucose transporters participate in uptake regulation of intracellular glucose^38^. Authors suggested that insulin has the ability to compress the paracellular barriers of the airway epithelium, creating an airway metabolite barrier against bacterial infections.

The inflammatory kinases JNK and IκK promote the phosphorylation of IRS-1 in serine 312 in humans and serine 307 in mice^39,40^, leading to insulin resistance by interrupting IR-β/IRS interaction^41^ and stimulating IRS-1 degradation^39^. The cytokine TNF-α, via activation of JNK and IκK, is reported to play an important role leading to impairment of insulin action^42,43^. In high-fat diet-fed mice, the TNF-α-mediated insulin resistance involves inhibition of the AKT/NO signaling^37^. Our study demonstrated that the phosphorylation of JNK and IκK are significantly elevated in lung tissue of obese mice, suggesting that an inflammatory response could mediate the impairment in insulin signaling induced by high-fat diet. Interestingly, in non-obese OVA-challenged mice, insulin-induced AKT phosphorylation was partly (despite significantly) reduced in comparison non-challenged group, indicating a role for the airway inflammation by itself on the impaired insulin signaling. TNF-α has been implicated in asthma physiopathology^44^, and anti-TNF-α antibody attenuates the aggravation of allergic airway inflammation in obese mice^10^, strongly suggesting that this cytokine may be a link between asthma and insulin impaired in the high-fat diet-fed mice.

The iNOS enzyme is highly expressed in lungs of asthmatic patients and animals, generating high levels of NO in the exhaled air^3^. Activation of NF-kB by TNF-α increases iNOS transcription, leading to subsequent NO overproduction^45^, which favors the eosinophil mobilization to the lungs^5^. Therefore, the NO pathway seems to provide experimental support for the elucidation of the association between asthma and impaired insulin signaling. An early study showed that iNOS expression is markedly increased in mice fed with a high-fat diet in mice^12^. Further studies demonstrated that either chronic NO inhibition or deletion of the iNOS gene in high-fat diet-fed mice decreases the adiposity and adipose tissue inflammation, and improves insulin signaling in skeletal muscle thus suggesting a role for NO in the development of obesity-associated insulin resistance^13,46^. As a mechanism, it was shown that excess of NO could cause a nitrosative stress in skeletal muscle hallmarked by covalent modification and inhibition of key proteins of the insulin signaling^5,16^. To evaluate if a nitrosative stress response was taking place in the lungs of obese mice, we carried out immunoprecipitation and immunofluorescence assays. The immunoprecipitation assay was used to more specifically study the nitrated proteins in our samples, and the immunofluorescence assay was used to confirm the occurrence of protein nitration in the lungs. We found that the reduction of insulin-stimulated kinase activity (phosphorylation) lung tissue of obese mice was accompanied by an increased nitration of tyrosine residues of AKT, IRS-1 and IRβ as compared with lean mice. Immunofluorescence staining data confirmed an increased tyrosine nitration of AKT and IRβ in obese compared with lean mice.

Aminoguanidine inhibits both iNOS activity and expression, and reduces nitrosative stress^47^. We thought that iNOS-derived NO was implicated in the impaired insulin transmission in the lungs of obese mice by nitrosative-dependent mechanisms. To test this hypothesis, obese and lean mice were treated with the iNOS inhibitor aminoguanidine, after which the phosphorylation of insulin signaling proteins in the lung tissues was evaluated. Our data showed that aminoguanidine treatment in obese mice restored the insulin-stimulated phosphorylation of AKT, IRS-1 and IRβ, and abrogated the increase in JNK and IκK phosphorylation in lung tissue. Therefore, these data seem to confirm the strong association between the nitrosative pathways with the impaired insulin signaling in the lungs. Hyperglycemia in diabetes is indirectly associated with overproduction of reactive-oxygen species (ROS) through the formation of advanced glycated end-products (AGE)^48^. Aminoguanidine given at high doses (approximately 1 g/kg/day) in hyperglycemic rodent is reported to inhibit AGE generation^49^. In our study, the dose used of aminoguanidine was 50 times lower (20 mg/kg/day), which is suggestive that reversal of the insulin signaling impairment by this compound in obese mice takes place independently of this action.

The polyphenol resveratrol has been thought as an adjuvant therapy in many cases. Resveratrol improves glucose intolerance and insulin resistance^50^. We showed recently that resveratrol treatment in high-fat diet-fed obese mice exerts an anti-inflammatory in the lungs of obese mice, as evaluated by reductions of OVA-induced pulmonary eosinophil infiltration, iNOS expression and TNF-α levels^24^. Reduced p47phox expression and ROS levels by resveratrol in lungs of OVA-challenged obese mice were also reported^24^. We thought therefore that the protective actions of resveratrol in the allergic airway inflammation of obese mice could at least partly reflect the restoration of the insulin signaling. Our data showed that two-week therapy of obese mice with resveratrol restored the phosphorylation of IRS, IRβ and AKT and abrogated the phosphorylation of JNK and IκK. Data of immunoprecipitation and immunofluorescence assays also showed that resveratrol treatment reverses the increased nitration of tyrosine residues in AKT, IRβ and IRS-1 in lungs of obese mice. Therefore, it is reasonable to assume that the amelioration of asthma exacerbations in insulin resistant obese mice by resveratrol may be due to restoration of the insulin signaling pathway in the lungs. Consistent with our data, in the mouse liver of high-fat diet-fed mice, resveratrol therapy was shown to restore the phosphorylation levels of different proteins involved in the insulin signaling pathway such as Akt, IRS-1, PI-3K, PDK-1, and GSK-3^51^.

Loss weight in patients undergoing bariatric surgery or low-calorie-diet leads to improvements of several markers of asthma^52^. In our study, resveratrol-treated obese mice had a small but significant weight loss (as demonstrated by the reductions body weight and epididymal fat mass), which is consistent with previous studies^24,51,53^. Therefore, we may not exclude that amelioration of the insulin signaling transmission by resveratrol in obese mice reflects at least in part the reduction of body measures.

In summary, we report here that lungs of obese mice display nitrosative-associated impairment of insulin signaling, which may amplify asthma exacerbations associated with obesity. Treatment with resveratrol reestablished the insulin signaling pathway in the lungs of obese mice, placing this compound as a putative pharmacological strategy aiming to reduce the asthma exacerbations in obese individuals.

## Materials and Methods

### Animals and high-fat diet

All animal procedures and experimental protocols were approved by the Institutional Committee for Ethics in Animal Use (CEUA) of State University of Campinas (UNICAMP; protocols number 3501-1 and 3502-1). All experiments were performed in accordance with relevant guidelines and regulations. Male C57BL6/J mice (3-week old) were provided by the Central Animal House Services, and housed 3 per cage at a constant room temperature under a 12 hour light/dark cycle. By reaching 4 weeks of age, the animals were fed for additional12 weeks with a high-fat diet to induce obesity (carbohydrate: 29%; protein: 16%; fat: 55%). The control group (lean mice) received standard chow (carbohydrate: 70%; protein: 20%; fat: 10%). By reaching 16 weeks of age, mice were used for experimentation^8^.

### Drug treatments

Lean and obese mice were treated with resveratrol (Sigma-Aldrich Co., St. Louis, MO) dissolved in distilled water at 100 mg/kg/day, given by gavage during the 15^th^ and the 16^th^ weeks of life^54^. An independent set of lean and obese mice were treated with the iNOS inhibitor aminoguanidine (20 mg/kg/day; Sigma-Aldrich Co., St. Louis, MO) given in the drinking water during the 14^th^, the 15^th^ and the 16^th^ weeks of life^9^.

### Sensitization and challenge with ovalbumin (OVA)

At the 14^th^ week, lean and obese mice were sensitized with a subcutaneous injection (0.4 ml) of 100 μg of OVA (Grade V; Sigma-Aldrich Co., St. Louis, MO) mixed with 1.6 mg Al(OH)_3_ in 0.9% NaCl. One week later (15^th^ week), mice received a second subcutaneous injection of 100 μg OVA (0.4 ml). On the 16^th^ week, animals were intranasally challenged with OVA (10 μg/50 μl) twice a day for two days. Control non-challenged mice were instilled with saline (50 μl) instead of OVA. At 48 h after the first OVA-challenge (or saline instillation), animals were anaesthetized through isoflurane inhalation and cervical dislocation, and lungs and/or isolated bronchi were collected for Western blot, immunoprecipitation and immunofluorescent studies, as detailed below. In all groups, mice (lean and obese) were used after 6 h of fasting.

### Experimental designs and insulin stimulation

In the experimental protocols designed to investigate the whole lung tissue, the left lung was removed *in situ* and immersed immediately in extraction buffer (10% sodium dodecyl sulfate, 100 mM Tris, 10 mM ethylenediaminetetraacetic acid [EDTA], 10 mM sodium pyrophosphate, 100 mM sodium fluoride, 10 mM sodium vanadate, pH 7.4). Lung tissue (0.1 g) was homogenized using a Polytron PTA 20 S generator (model PT 10/35; Brinkmann Instruments, Inc., Westbury, NY). For the samples comprising the insulin-induced phosphorylation of AKT and IRS-1 and IRβ, mice received an intravenous injection of insulin (1U per animal; 0.1 ml) via the inferior vena cava and the lungs were removed within 5 min after injection^55^. In the experimental protocols designed to investigate the isolated bronchi, anaesthetized mice (stimulated or not with 1U of insulin) were killed by cervical dislocation. Lungs were dissected free as a block and immediately placed in a Petri dish containing Krebs-Henseleit solution (mM: 117 NaCl, 4.7 KCl, 2.5 CaCl_2_, 1.2 MgSO_4_, 1.2 KH_2_PO_4_, 25 NaHCO_3_ and 11 glucose). The left and right main bronchi were then carefully removed and approximately 2 mm rings were excised and immersed in the extraction buffer. Samples were stored at −80 °C until processed for the western blotting assays for p-AKT.

### Western blotting and immunoprecipitation analysis

Samples from lung tissue and isolated bronchi were centrifuged (12,000 × *g*, 20 min, 4 °C) and the supernatant had the protein concentration determined by Bradford Assay (BioRad, Hercules, CA, USA). Equal amount of proteins were resolved by 4–15% SDS-PAGE and transferred to nitrocellulose membranes. After blocking with 5% nonfat dried milk, the membranes were incubated with primary antibodies against p-IRS-1 Tyr 632 (sc-17196), p-IRβ Tyr 1162/1163 (sc-25103), p-AKT Ser 473 (sc- 7985-R), p-JNK 1/2/3 (sc-135642) and p-IκK (sc-23470-R), nitrotyrosine (39B6) (sc- 32757) (Santa Cruz Biotechnology, Santa Cruz, CA, USA) or iNOS (ab15323) (AbCam Technology, Cambridge, UK). Next, membranes were allowed to react with horseradish peroxidase-conjugated secondary antibody (BD Biosciences, San Diego, CA, USA) at room temperature for 90 min using a Western Blot Enhanced Chemiluminescence (ECL) method. The protein bands were visualized by ChemiDoc and analyzed with the ImageLab Software (Version 5.2.1; Bio Rad Laboratories, Hercules, CA, USA). To examine the nitration in tyrosine residues, we performed immunoprecipitation assay in lung tissue. Briefly, supernatant of the lung samples containing equal amount of proteins were incubated with antibody against nitrotyrosine (Santa Cruz Biotechnology, Santa Cruz, CA, USA) for 2 h, and subsequently incubated with protein A Sepharose overnight at 4 °C. Immune complex was washed three times in PBS (pH 7.4) containing 1% NP-40 and 2 mM Na_3_VO_4_, resuspended in Laemmli buffer and boiled for 5 min. The immunecomplexes were submitted to SDS-PAGE and processed for western blotting analysis for AKT-tyrosine, IRβ-tyrosine and IRS-1-tyrosine. Results are represented as the ratio of the density of the primary antibodies band to the density of the β-actin band.

### Immunofluorescence staining

Lung tissues were washed twice with PBS, fixed with 3.5% paraformaldehyde for 15 min at room temperature and permeabilized with 0.1% Triton X-100 in PBS. Paraffin embedded 5-micron sections of all the tissue samples. Briefly, the tissue sections were deparaffinized in two changes of xylene for 5 min each, hydrated in two changes of 100% ethanol for 3 min each, changes of 95% and 80% ethanol for 1 min each, and finally washed in distilled water. The sections were processed for antigen retrieval in a water bath containing sodium citrate buffer (10 mM citric acid, 0.05% Tween 20, pH 6.0) at 95–100 °C for 40 min, and then allowed to cool to room temperature for another 60 min. The sections were rinsed in PBS-Tween 20, twice for 2 min each. The sections were blocked with 2% BSA in PBS for 1 h at room temperature. The sections were then incubated with a 1:500 dilution of the homologous primary N-specific antiserum 3-NT (39B6; sc- 32757), IRβ (C-19; sc-711) and AKT (sc- 8312) in PBS for 1 h in a humidified chamber. After three washes in PBS, the sections were incubated with the secondary antibody rhodamine (sc-2095) or fluorescein (sc- 2989) for 30 min. After a further wash cycle, the sections were mounted with 4,6-diamidino-2-phenylindole (DAPI) and viewed under an immunofluorescence microscope (Leica DM 4500 B).

### Data Analysis

All data are presented as means ± SEM. The effects of the treatments were compared by ANOVA analysis followed by Tukey post hoc test. Differences were considered to be statistically significant at p < 0.05.

### Data Availability

The datasets generated during and analysed during the current study are available from the corresponding author on reasonable request.

## Electronic supplementary material

Supplementary information
