# Supplementary material for: High-fat diet-induced obesity impairs insulin signaling in lungs of allergen-challenged mice: Improvement by resveratrol
Source: Sci Rep. 2017 Dec 11;7:17296. doi: 10.1038/s41598-017-17558-w (PMC5725490; doi:10.1038/s41598-017-17558-w)
Supplement: Supplementary file 1 — Supplementary information [file 41598_2017_17558_MOESM1_ESM.pdf]

High-fat diet-induced obesity impairs insulin signaling in lungs of allergen-challenged mice: Improvement by resveratrol

Diana M. André, Marina C. Calixto, Carolina Sollon, Eduardo C. Alexandre, Edith B. G. Tavares, Ana C. A. Naime, Gabriel F. Anhê & Edson Antunes

Department of Pharmacology, Faculty of Medical Sciences, University of Campinas (UNICAMP), Campinas, São Paulo, Brazil

Supplementary Figure S1: Length gels relative to Fig 1A, B and C. Red boxes indicate the cropping lines used to generate the figures.

A.

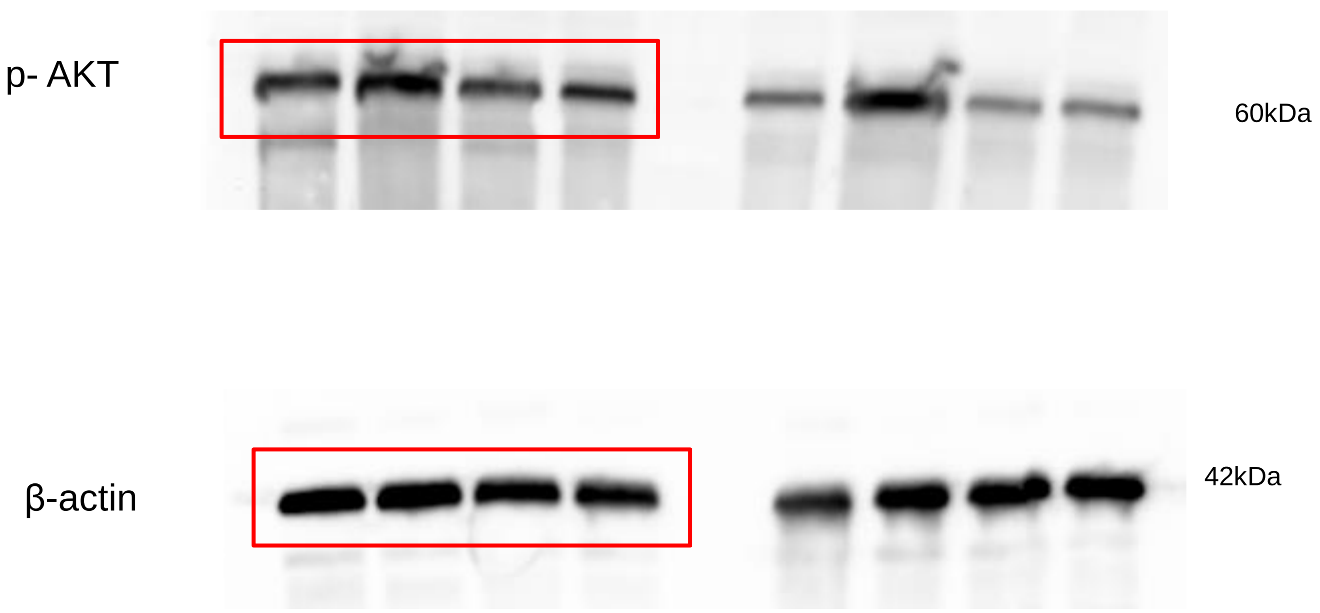

Supplementary Figure S1

B.

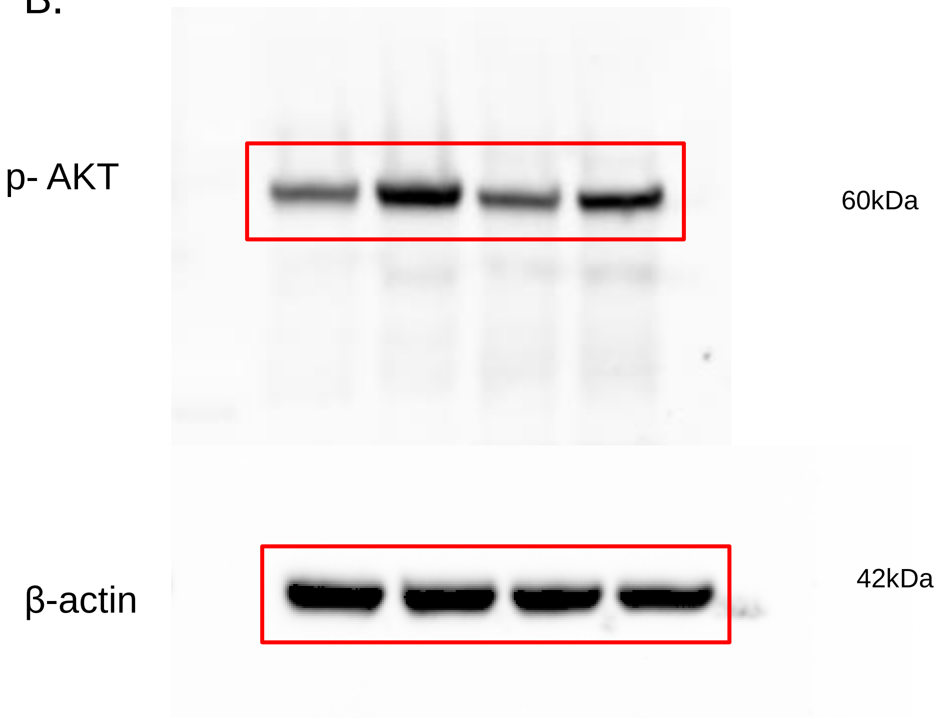

Supplementary Figure S1

C.

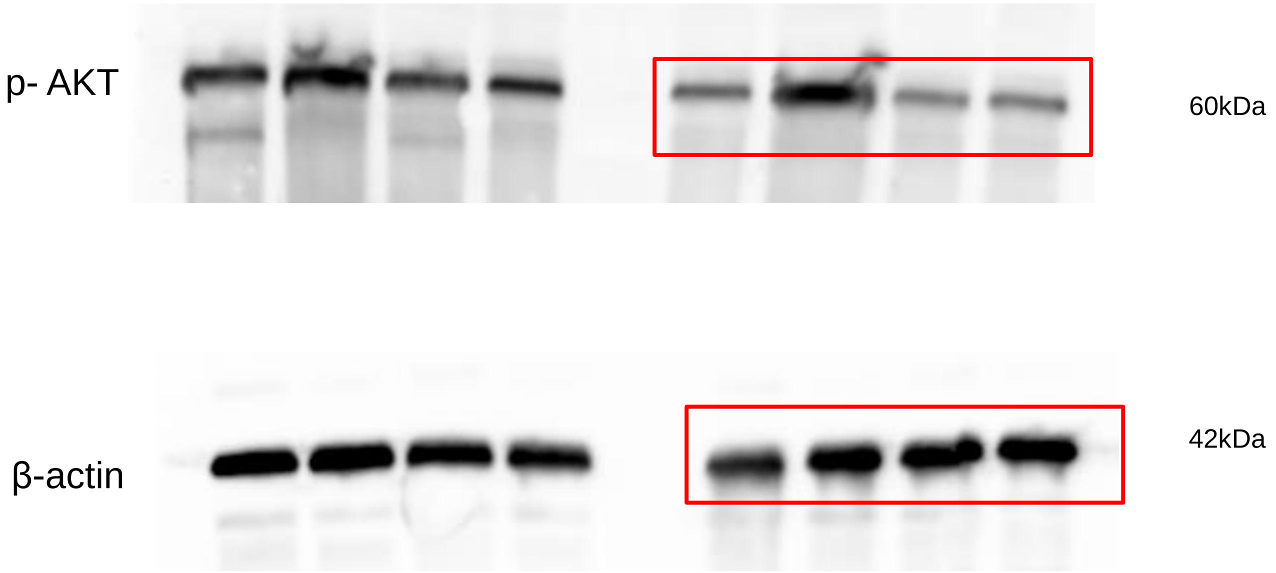

Supplementary Figure S2: Length gels relative to Fig 2A, B, C and D. Red boxes indicate the cropping lines used to generate the figures.

A.

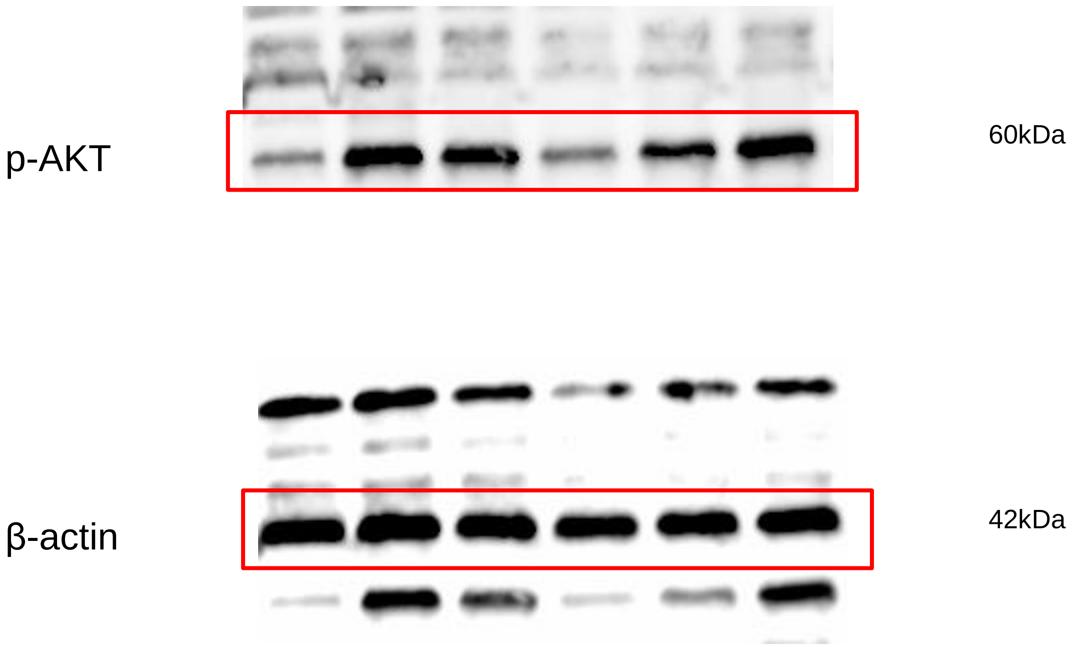

Supplementary Figure S2

B.

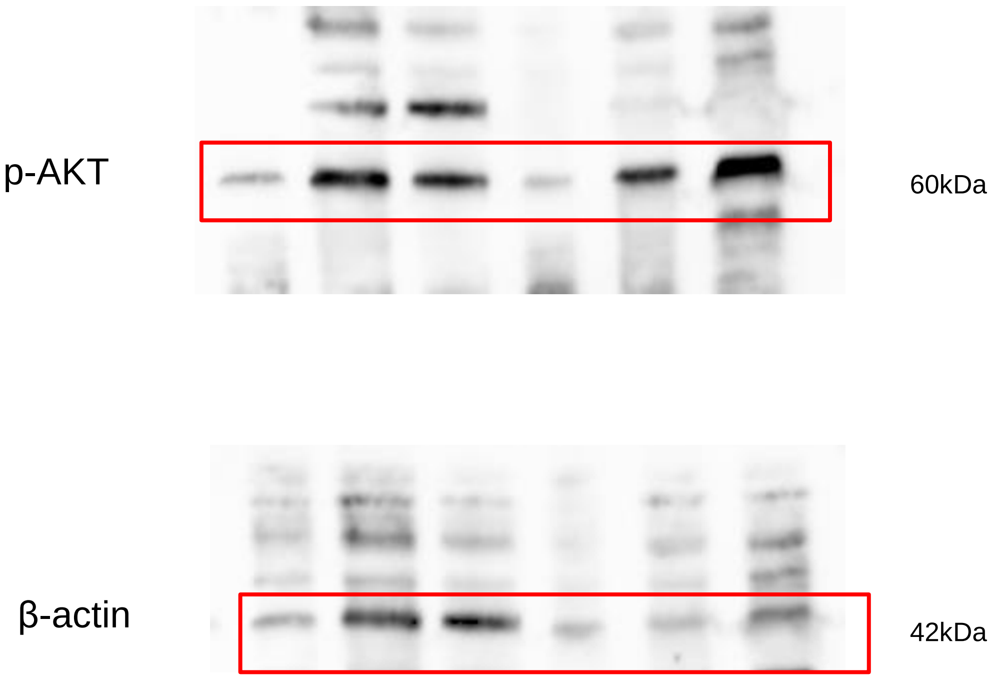

Supplementary Figure S2

C.

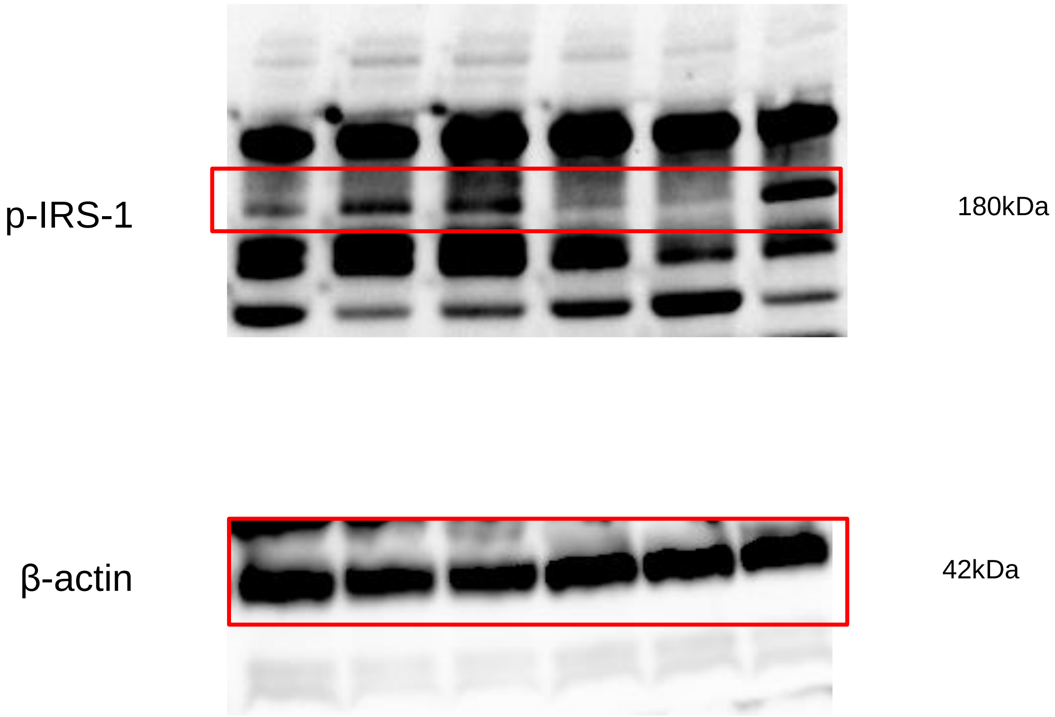

Supplementary Figure S2

D.

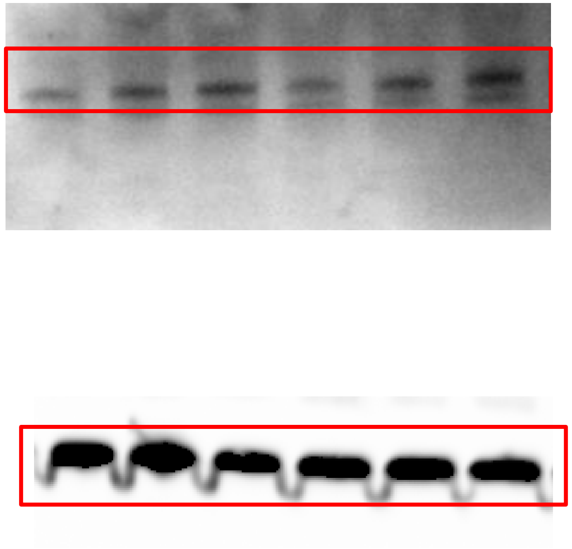

Supplementary Figure S3: Length gels relative to Fig 3A, B and C. Red boxes indicate the cropping lines used to generate the figures.

A.

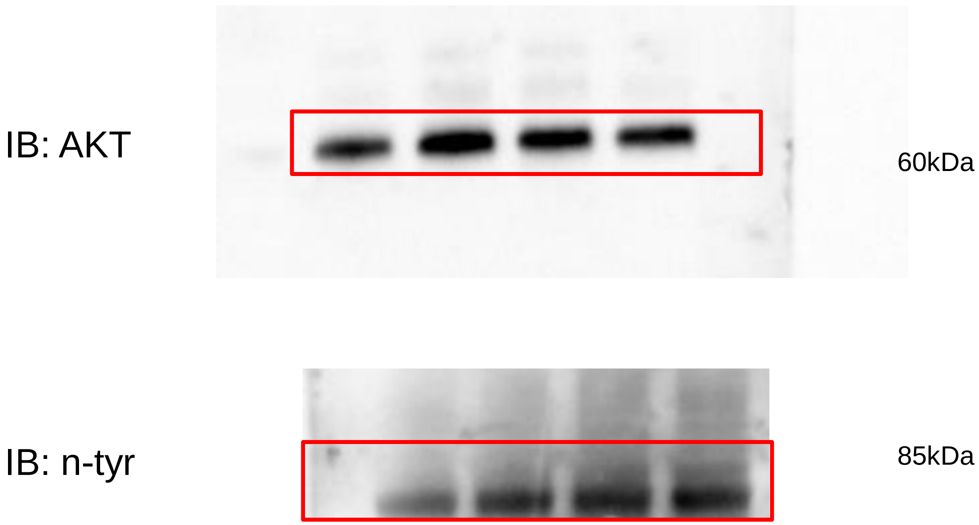

Supplementary Figure S3

B.

IB: IRS-1

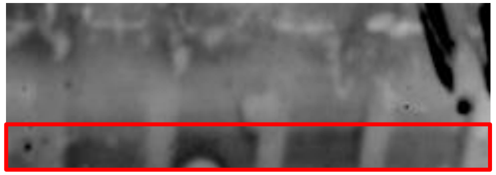

180kDa

IB: n-tyr

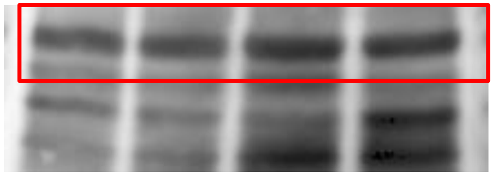

85kDa

Supplementary Figure S3

C.

IB: IRβ

95kDa

IB: n-tyr

85kDa

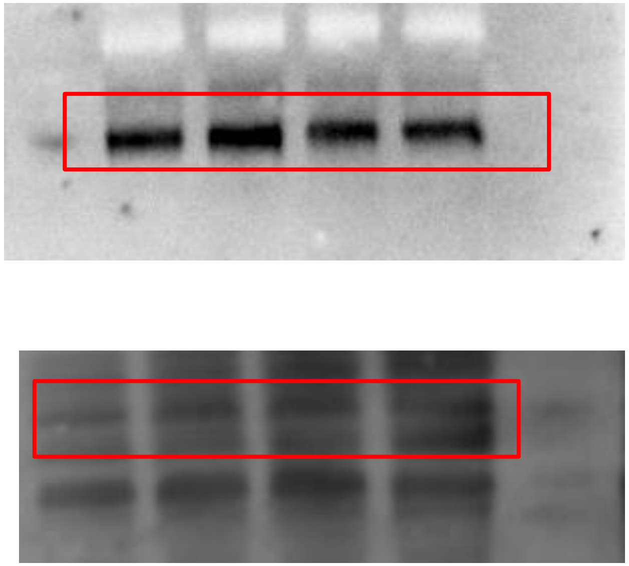

Supplementary Figure S6: Length gels relative to Fig 6A and B. Red boxes indicate the cropping lines used to generate the figures.

A.

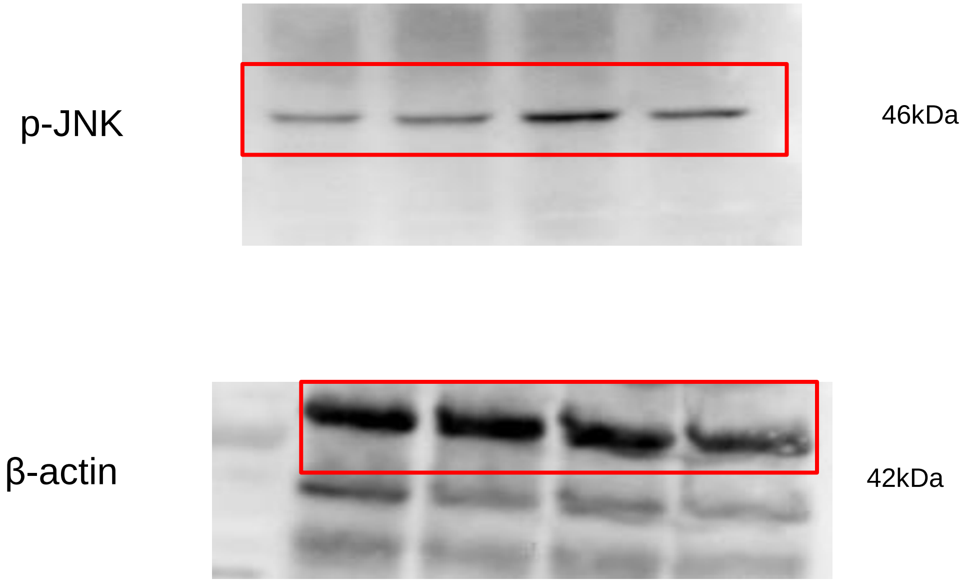

Supplementary Figure S6

B.

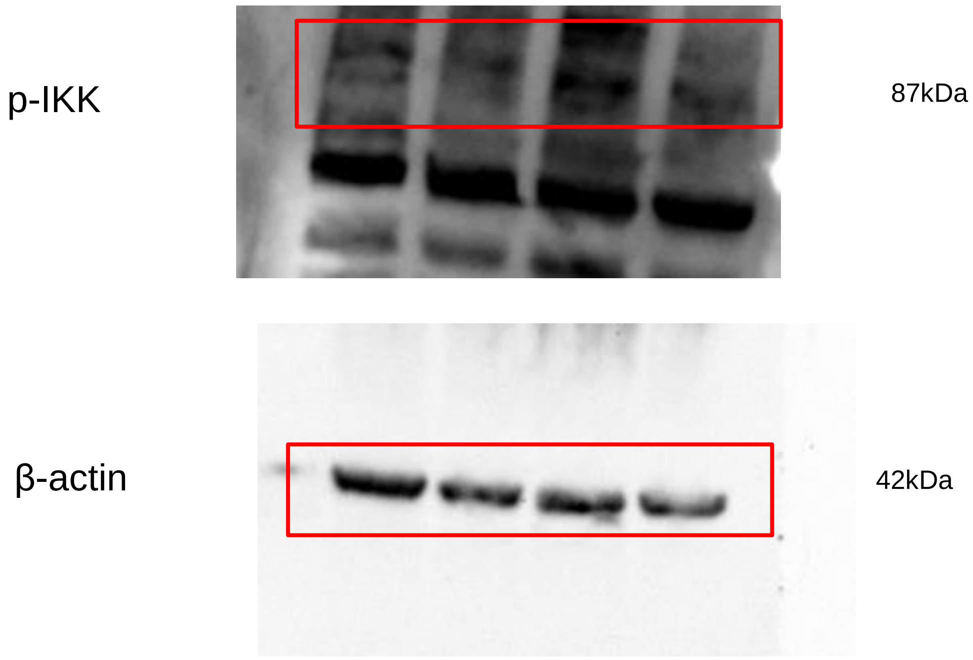

Supplementary Figure S7: Length gels relative to Fig 7A, B, C and D. Red boxes indicate the cropping lines used to generate the figures.

A.

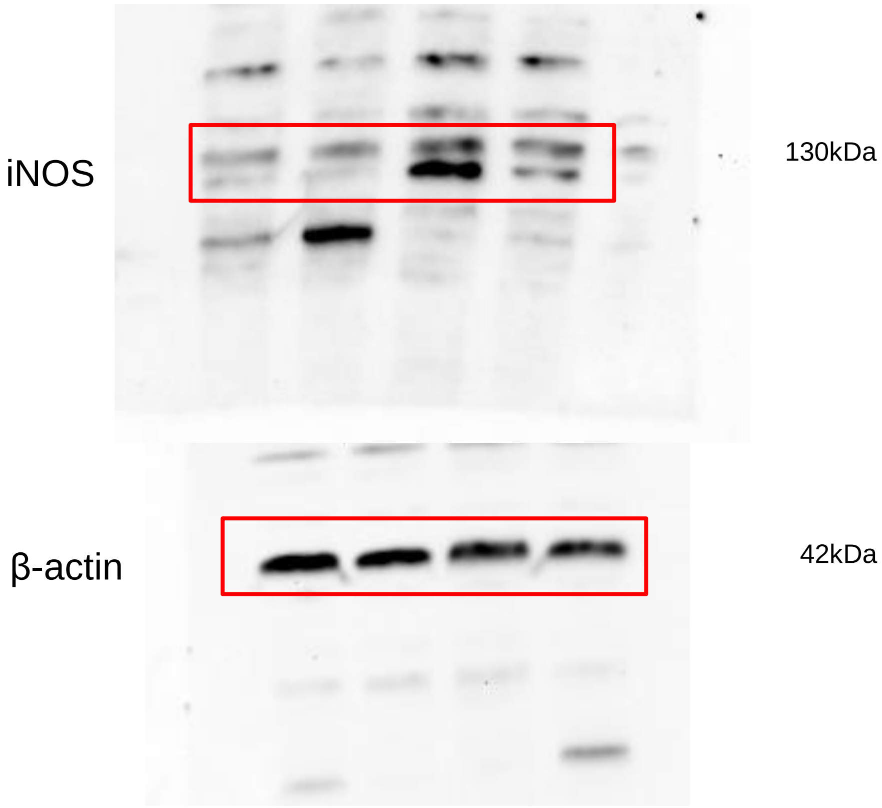

Supplementary Figure S7

B.

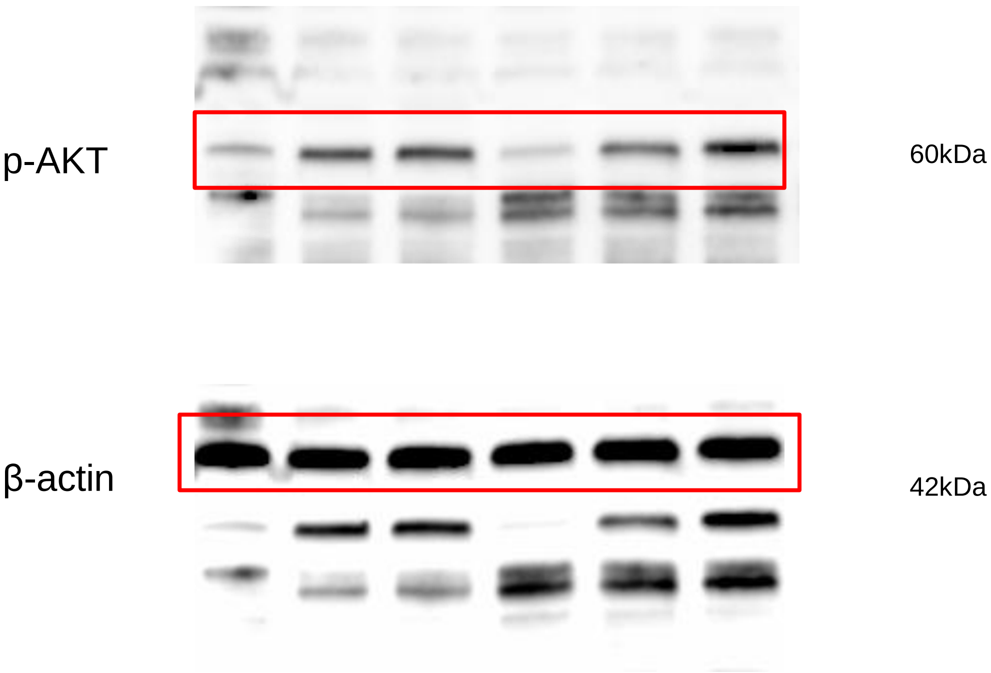

Supplementary Figure S7

C.

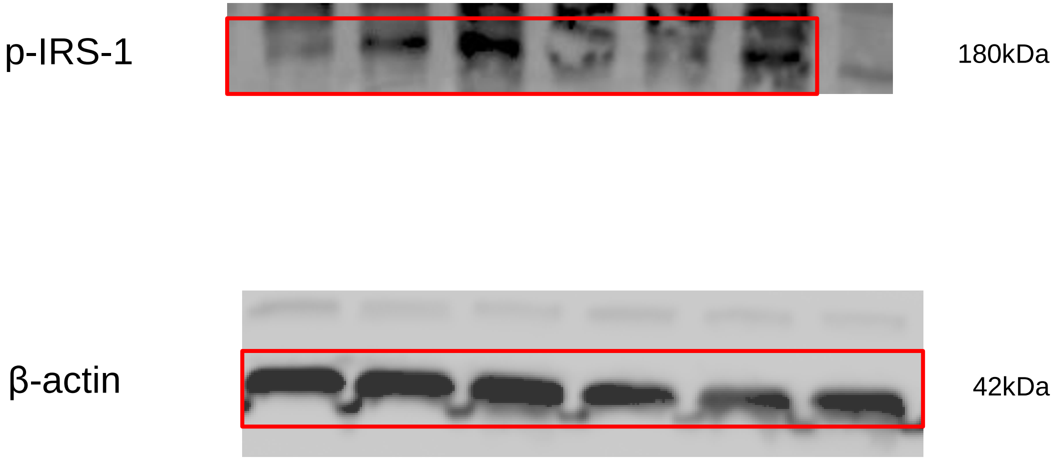

Supplementary Figure S7

D.

p-IRβ

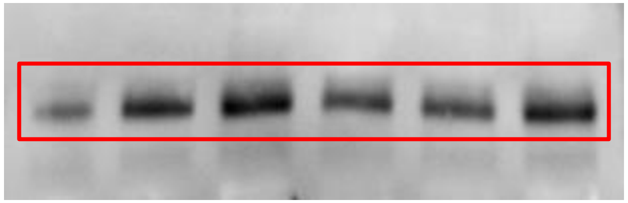

95kDa

β-actin

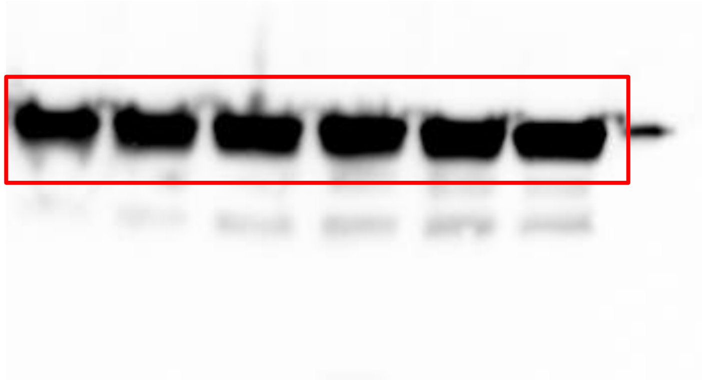

42kDa

Supplementary Figure S8: Length gels relative to Fig 8A and B. Red boxes indicate the cropping lines used to generate the figures.

A.

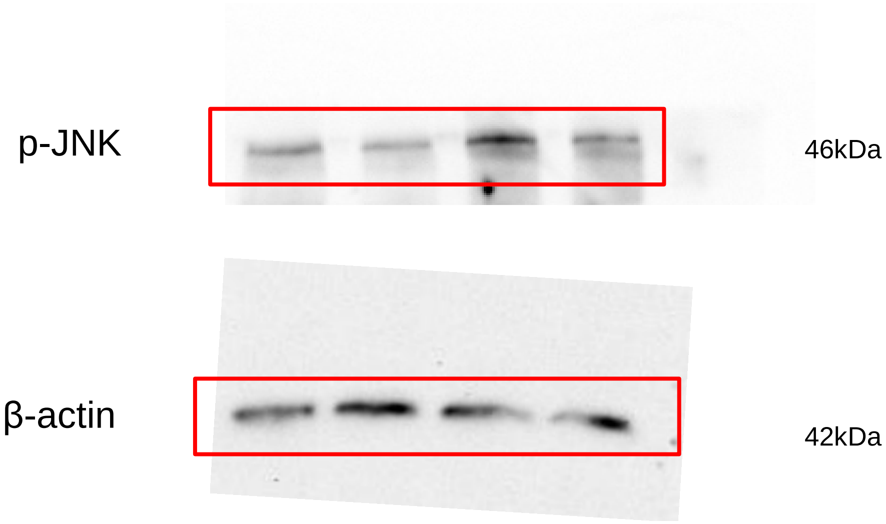

Supplementary Figure S8

B.

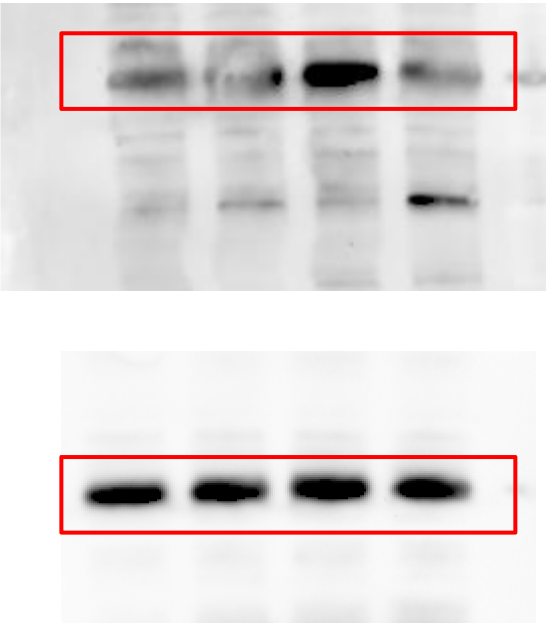

p-IKK 87kDa

β-actin 42kDa
